# Integrated genomic epidemiology and phenotypic profiling of *Clostridium difficile* across intra-hospital and community populations in Colombia

**DOI:** 10.1038/s41598-019-47688-2

**Published:** 2019-08-05

**Authors:** Marina Muñoz, Daniel Restrepo-Montoya, Nitin Kumar, Gregorio Iraola, Milena Camargo, Diana Díaz-Arévalo, Nelly S. Roa-Molina, Mayra A. Tellez, Giovanny Herrera, Dora I. Ríos-Chaparro, Claudia Birchenall, Darío Pinilla, Juan M. Pardo-Oviedo, Giovanni Rodríguez-Leguizamón, Diego F. Josa, Trevor D. Lawley, Manuel A. Patarroyo, Juan David Ramírez

**Affiliations:** 1 Grupo de Investigaciones Microbiológicas–UR (GIMUR), Departamento de Biología, Facultad de Ciencias Naturales y Matemáticas, Universidad del Rosario, Bogotá, Colombia; 2 Posgrado Interfacultades Doctorado en Biotecnología, Facultad de Ciencias, Universidad Nacional de Colombia, Bogotá, Colombia; 3 Genomics and Bioinformatics Department, North Dakota State University, Fargo, North Dakota USA; 4 Host-Microbiota Interactions Laboratory, Wellcome Sanger Institute, Hinxton, UK; 5 Microbial Genomics Laboratory, Institut Pasteur Montevideo, Montevideo, Uruguay; 6 Center for Integrative Biology, Universidad Mayor, Santiago de Chile, Chile; 7 Molecular Biology and Immunology Department, Fundación Instituto de Inmunología de Colombia (FIDIC), Bogotá, Colombia; 8 School of Medicine and Health Sciences, Universidad del Rosario, Bogotá, Colombia; 9 Faculty of Animal Sciences, Universidad de Ciencias Aplicadas y Ambientales (UDCA), Bogotá, Colombia; 10 Hygea group, Faculty of Health Sciences, Universidad de Boyacá, Tunja, Colombia; 11 Centro de Investigaciones Odontológicas, Facultad de Odontología, Pontificia Universidad Javeriana, Bogotá, Colombia; 12 PhD Programme in Biomedical and Biological Sciences, Faculty of Natural Sciences and Mathematics/School of Medicine and Health Sciences, Universidad del Rosario, Bogotá, Colombia; 13 Hospital Universitario Mayor – Méderi, Universidad del Rosario, Bogotá, Colombia; 14 Fundación Clínica Shaio, Bogotá, Colombia

**Keywords:** Bacteriology, Bacterial genes

## Abstract

*Clostridium difficile*, the causal agent of antibiotic-associated diarrhea, has a complex epidemiology poorly studied in Latin America. We performed a robust genomic and phenotypic profiling of 53 *C*. *difficile* clinical isolates established from diarrheal samples from either intrahospital (IH) or community (CO) populations in central Colombia. *In vitro* tests were conducted to evaluate the cytopathic effect, the minimum inhibitory concentration of ten antimicrobial agents, the sporulation efficiency and the colony forming ability. Eleven different sequence types (STs) were found, the majority present individually in each sample, however in three samples two different STs were isolated. Interestingly, CO patients were infected with STs associated with hypervirulent strains (ST-1 in Clade-2). Three coexistence events (two STs simultaneously detected in the same sample) were observed always involving ST-8 from Clade-1. A total of 2,502 genes were present in 99% of the isolates with 95% of identity or more, it represents a core genome of 28.6% of the 8,735 total genes identified in the set of genomes. A high cytopathic effect was observed for the isolates positive for the two main toxins but negative for binary toxin (TcdA+/TcdB+/CDT− toxin production type), found only in Clade-1. Molecular markers conferring resistance to fluoroquinolones (*cdeA* and *gyrA*) and to sulfonamides (*folP*) were the most frequent in the analyzed genomes. In addition, 15 other markers were found mostly in Clade-2 isolates. These results highlight the regional differences that *C*. *difficile* isolates display, being in this case the CO isolates the ones having a greater number of accessory genes and virulence-associated factors.

## Introduction

*Clostridium difficile* (CD) is an anaerobic Gram-positive spore-forming bacillus recognized as the causal agent of antibiotic-associated diarrhea and a wide range of gastrointestinal syndromes, including pseudomembranous colitis and toxic megacolon, that in complex cases can result in death^1^. The impact of *C*. *difficile* infection (CDI) has been well documented in North America, Europe, and some regions of Asia, especially at the intrahospital (IH) level where individuals are exposed to the main risk factors for *C*. *difficile* proliferation, among them, antibiotic therapy^2^. In Latin America, studies focused on determining the frequency of CDI and identifying the hypervirulent strain RT027/BI/NAP1 (belonging to ST-1, Clade 2) have been carried out mainly in Argentina, Colombia, Brazil, Costa Rica, Chile and Perú^3^.

*C*. *difficile* features two main toxins (ToxA and ToxB), which belong to a large family of clostridial toxins with glucosyltransferase activity^4^ that are responsible for the presentation of symptoms caused by damage to the epithelial tissue of the infected host^5^. Besides the main toxins, the binary toxin (composed of subunits *CdtA* and *CdtB*), which has ADP-ribosyl transferase activity, may have a role in the adherence of *C*. *difficile* by acting in a synergistic way with other factors such as surface proteins^6^. Another factor involved in the complexity of CDI management is antibiotic resistance, which has been associated with polymorphisms and/or the presence of genes that can be transported by mobile genetic elements^7^. Additionally, the ability of *C*. *difficile* to sporulate and germinate (aspects that impact on the dissemination and colonization of the microorganism), as well as to present resistance to certain antimicrobials (which affects the response to treatment), contribute significantly to the impact of *C*. *difficile* in their hosts^8^.

Whole-genome sequencing of *C*. *difficile* is a prerequisite to understand the molecular and genomic basis of the phenotype diversity involved in the wide range of clinical impacts this microorganism can cause. A detailed high-resolution genomic and phenotypic profiling of *C*. *difficile* samples collected in Bogotá (central Colombia) was thus carried out here, to obtain a broad insight about differences in CDI in community and intrahospital populations in Bogotá, Colombia.

## Results

### Genome sequencing, assembly, and species allocation

Fifty-three clinical isolates were established from 17 fecal samples from patients suffering diarrhea in whom CDI was detected as part of a previous screening^9^. A variable number of isolates were recovered for each sample (1 to 5), as a strategy to recover multiple genotypes potentially coexisting in a same sample as reported elsewhere^10^. Isolates information is described in Supplementary Table S1. The complete set of isolates was subjected to genomic and phenotypic characterization as is described in Supplementary Fig. S1. Thirty of the isolates (56.6%) were from the Fundación Clínica Shaio (Bogotá, Colombia) and the remaining 23 (43.4%) were from the Hospital Universitario Mayor – Méderi (Bogotá, Colombia). The 60.4% (*n* = 32) of the total isolates corresponded to health-care associated infections, collected at intra-hospital ‘IH’ services, and the remaining 39.6% were community-acquired infections (CO) (*n* = 21).

The analyzed genomes contained between 4.1 and 4.4 mega bases, with an average N50 of 422,610 nt (range 156,209–857,412 nt) for the assemblies and a depth of at least 319.3 × (Supplementary Data set S1). The species allocation of the genomes was verified using the average identity of nucleotides (ANI)^11^ and considering ANI > 95.0 as the cutoff point.

### *C*. *difficile* Clades 1 and 2 and coexistence of multiple sequence types were found within Colombian samples

**Figure 1:**
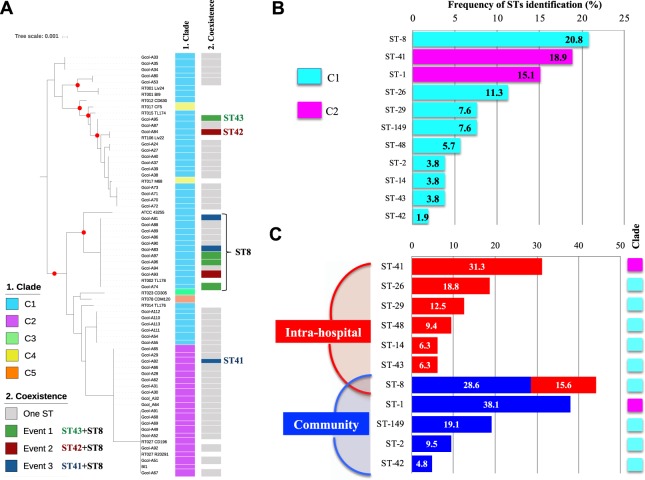
Multilocus sequence typing analysis of the whole genome sequences of the *Clostridium difficile* isolates. (**A**) Frequency of sequence type (ST) identification by MLST clade in the studied isolates (*n* = 53). (**B**) Frequency of ST identification by population: intrahospital (IH in red; *n* = 32) and community (CO in blue; *n* = 21). (**C**) Phylogenetic reconstruction from the alignment of the concatenated sequences of the seven genes used for MLST. The red dots indicate well-supported clusters (bootstrap ≥90.0%). The coexistence of ST events, defined as the simultaneous identification of two STs in isolates established from the same patient, are marked on the right.

A total of 11 sequence types (STs) were identified in the set of isolates evaluated. Even though MLST phylogenetics could be an incomplete method, this is an important scheme for grouping isolates and has been the accepted strategy for intra-species classification for *C*. *difficile*^12^. Therefore, it was used as a first step to identify the clades to which circulating STs belong in the analyzed populations. All Colombian isolates are close to Clade-1 and Clade-2 members (Fig. 1A). Nevertheless, no independent grouping of the other clades reference sequences included in the analysis was observed. Such is the case of RT017_M68 and RT017_CF5 that despite belonging to Clade-3 are being grouped with Clade-1 strains.

In terms of ST diversity by Clade (Fig. 1B), 35 of the 53 isolates (66.0%) were in Clade-1, which contained 9 STs with ST-8 being the most abundant (20.8%), followed by ST-26 (11.3%). The remaining 18 isolates (34.0%) were grouped in Clade-2, which contained only 2 STs, ST-41 (18.9%) and ST-1 (15.1%). The discrimination of the STs by population (Fig. 1C) showed that 7 STs were found at the IH level, while 5 STs at the CO level. ST-8 (Clade-1 member) was the only ST found in both populations (IH and CO). STs belonging to Clade-2 occupied the first place in frequency for each population, being ST-1 (associated with hypervirulent strains) present at the CO population only.

In 14 of the 17 samples analyzed, a single ST was identified for all isolates established, showing that many of the isolates are highly clonal. However, in three samples, two STs were identified simultaneously, being defined as ‘coexistence events’ (defined as events where two STs simultaneously detected in the same sample) (Fig. 1A right panel). Two interesting findings were identified in those coexistence events: (i) ST-8 was always present in all three events; and (ii) STs belonging to two different Clades were found in coexistence event 3 (ST-8 and ST-41 from Clade-1 and Clade-2, respectively).

### Clustering by clade and identification of high numbers of accessory genes by pangenome analysis

**Figure 2:**
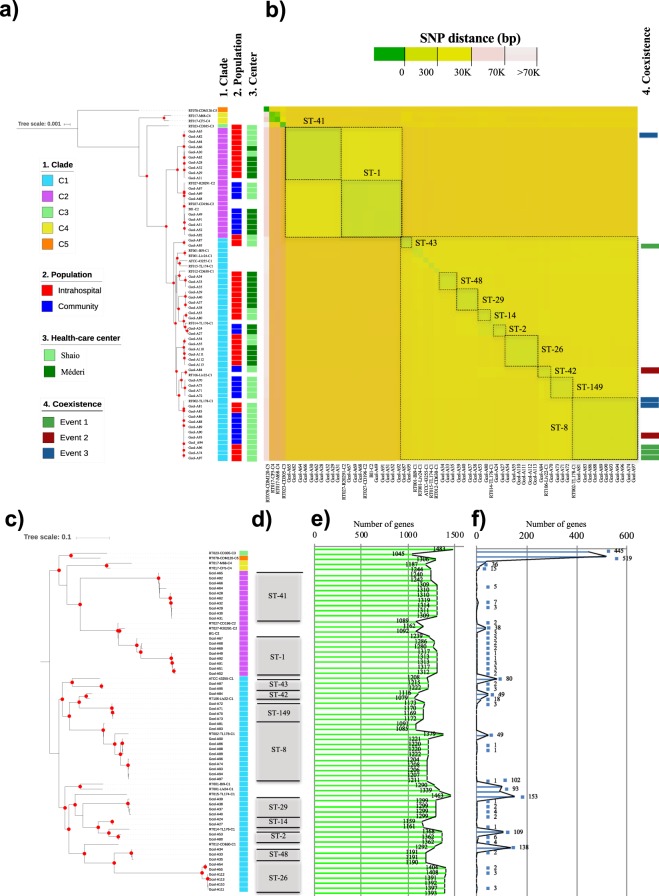
Determination of phylogenetic relationships from pangenome analysis. (**a**) Phylogenetic reconstruction based on the concatenated sequence of proteins that form the set of 2,502 core genes of the 53 Colombian clinical isolates analyzed plus 15 reference genomes. The sequences of the reference genomes of the clades typically described for *Clostridium difficile* (CD) were included in the analysis. Relationships by clade (1), population (2), and health care center (3) were constructed from this tree based on a core genome. (**b**) Pairwise comparison of core genome SNP distance between the evaluated genomes. Sequence types (STs) are marked on the core genome SNPs heatmap. (**c**) Phylogenetic reconstruction based on the concatenated sequence of the proteins that are part of the accessory genome of the isolates analyzed (*n* = 68). (**d**) Distribution of STs in the accessory genome phylogenetic reconstruction. (**e**) Number of accessory genes per analyzed genome. (**f**) Number of unique genes per genome. The coexistence events are marked on both the core genome SNP distance heatmap and accessory genome clustering. The red dots indicate well-supported clusters (bootstrap ≥90.0%). Nt, nucleotides; K, 1,000 nt.

A total of 8,735 gene clusters were identified and most of them belonged to the accessory genome (71.4%). The core genome was limited to only 2,502 genes, corresponding to 28.6% of the total genes found. The phylogenetic reconstruction based on 99,527 homologous positions of core genome had better discrimination than that based on MLST, which allowed independent clustering by clade of the genomes in each of the five clades (Fig. 2a). However, when the topology of the obtained tree was compared with the population origin (IH/CO) and with the hospital where the samples were collected (Méderi/Shaio), no origin-specific clustering was found. This confirmed that *C*. *difficile* isolates that belonged to the two clades were circulating in both populations and in the two hospital centers (Fig. 2a).

Single nucleotide polymorphism (SNP) distances was determined among isolates, from the core genome alignment (2,502 core genes). The results showed that isolates belonging to the same ST and established from the same sample carried from 0 to 71 SNPs (average: 15.5 SNPs per sample), being two samples collected from CO population those that transport the isolates with the highest number of SNPs into this group (71 and 69.3 in average with four and five isolates, respectively). An average of 215.6 SNPs was identified between isolates belonging to the same ST (although they have been isolated from different samples). Strikingly, ST-26 (in Clade-1) showed the highest SNP result (475.8 SNPs; Fig. 2b).

The accessory genome phylogenetic reconstruction revealed well-supported nodes with branches longer than those found for the core genome analysis (Fig. 2c). Although the topology of the tree was maintained, mainly in the members of Clade-2, there were some incongruences in the clustering of the other genomes (Fig. 2c). These incongruences included changes in the clustering of some STs, namely ST-42 and ST-43 as well as ST-26 and ST-48, which were now found to be closely related, the first two being involved in coexistence events (Fig. 2d). The new clustering in Clade-1 in the accessory genome tree may be associated with the high number of accessory genes identified in the analyzed genomes; at least 1,045 accessory genes per genome (Fig. 2e). There were also a high number of unique genes (Fig. 2f), especially in the genome of Gcol-A84 (established from a CO individual enrolled in Fundación Clínica Shaio), which had 49 unique genes and was one of the isolates showing changes in the tree clustering. Although some of the unique genes found in Gcol-84 corresponded to genes coding for proteins involved in biological processes or hypothetical/uncharacterized proteins, genes associated with antibiotic resistance and sporulation, as well as markers of mobile genetic elements were also found (Supplementary Table S2).

### Colombian isolates carry toxin coding genes with atypical organizations and have a cytopathic effect on Vero cells

Ariba analyses^13^ showed that 40 (75.5%) of the genomes contain toxin coding genes that were reported previously (Supplementary Fig. S2). Among them, *tcdA* (41.5%, *n* = 22) and *tcdB* (60.4%; *n* = 32), which code for the two main *C*. *difficile* toxins, are located within the pathogenicity locus (*PaLoc*), and *cdtA* and *cdtB* (34.0%; *n* = 18, present simultaneously), which encode for the subunits of the binary toxin, are located within the binary toxin (CDT) locus (*CdtLoc*). The toxigenic profiles of the isolates were analyzed according to the clade to which they belonged using a comparative approach. We found that none of the 18 isolates within Clade-2 (associated with hypervirulent strains) were positive for *tcdA*, only 10 (55.5%) were positive for *tcdB*, and the genes associated with the binary toxin (*cdtA* and *cdtB*) were detected exclusively in this clade.

**Figure 3:**
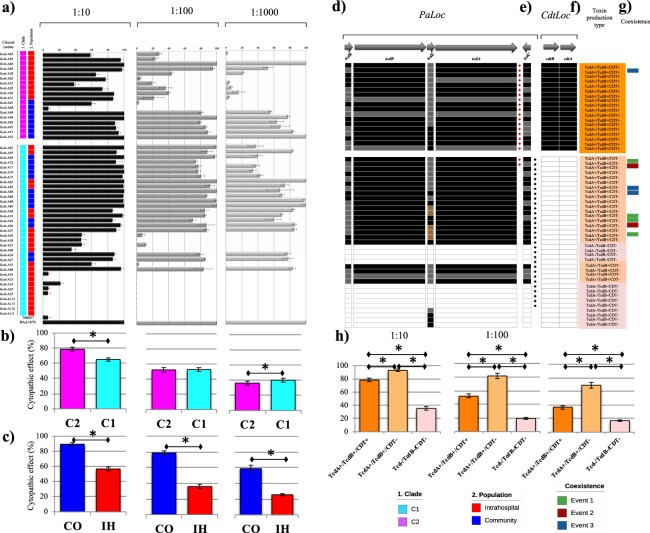
Phenotypic and genomic characterization of the toxigenic potential of the Colombian *Clostridium difficile* clinical isolates. (**a**) Cytopathic effect of culture supernatant by isolates in 1:10, 1:100, and 1:1000 dilutions on Vero cells, described in the context of phylogenetic reconstruction based on the accessory genome . (**b**) Cytopathic effect of culture supernatant per clade through the three dilutions evaluated. (**c**) Cytopathic effect of each culture supernatant per population. IH, intrahospital; CO, community. (**d**) Toxin coding genes located in the pathogenicity locus (*PaLoc*) identified by mapping against the *C*. *difficile* 630 reference strain. (**e**) Genes located in the CDT locus (*CdtLoc*) that encode binary toxin subunits. (**f**) Toxin production types (TPT) identified in the isolates studied. (**g**) Coexistence events in the isolates evaluated. (**h**) Cytopathic effect of culture supernatant according to the TPT identified. *The difference between means of positivity having statistically significant difference (p < 0.05).

The phenotypic tests aimed at evaluating the cytopathic effect of the culture supernatant in three dilutions (1:10, 1:100, and 1:1000) on Vero cells (a line of African green monkey kidney cells), were conducted according with the accessory genome clustering shown in Fig. 3c. This step of the phenotypical characterization revealed that only 9 isolates (17.0%) did not cause cell rounding, even at the highest evaluated concentration (1:10). These isolates showed an effect similar to that of the non-toxigenic reference *C*. *difficile* strain ATCC® 700057, which was included as the non-toxigenic control for the assay (Fig. 3a). The remaining 83.0% of the isolates (*n* = 44) caused cell rounding in more than 20% of the cells evaluated, including isolates Gcol-35, -37, -38, -39, and -40, in which toxin-coding genes were initially not detected (Supplementary Fig. S2). In general, although the isolates had a homogeneous effect between populations at the highest concentration (1:10), the effects were more stable through the dilutions in the established CO isolates than they were in the IH isolates where the cytotoxic effect was reduced in the 1:100 dilution and in the 1:1000 dilution (Fig. 3b,c). Further, the supernatants of isolates Gcol-52, -66, -81, and 89 caused the rounding of 100% of the cells even at the 1:1000 dilution, which was similar to the effect of the reference *C*. *difficile* strain ATCC® BAA-1870 that was evaluated as the toxigenic control of the assay (Fig. 3a). Although the comparative analysis by clade showed a significant increase in the mean cytopathic effect of the culture supernatant of the isolates in Clade-2 at 1:10 dilution, this difference was lost through the dilutions, being even higher in Clade-1 at dilution 1:1000, without any difference in the fold change per dilution (Fig. 3c). Interestingly, the comparison by population showed a marked differential pattern, with a significant increase in the mean cytopathic effect of the supernatants of the isolates established from CO and a clear difference in the fold change through the dilutions (Fig. 3d).

An exhaustive search of toxin coding genes in all the genomes analyzed was performed to clarify the genomic bases of this toxigenic effect, by mapping them against reference sequences of *PaLoc* and *CdtLoc*. The complete organization of these loci in the established isolates was analyzed by the clade to which they belonged (Fig. 3e,f). This exhaustive search showed that, when defining as positive any isolate that carried at least one gene previously associated with a toxigenic effect, the overall balance of toxigenic profiles increased to 83.0% (*n* = 44). This increase was accompanied by an increase in the overall frequency of detection of *tcdA* and *tcdB* to 71.7% (*n* = 38), these markers being present simultaneously and identified as positives in 100% of the Clade-2 isolates. The traditional toxin production types (TPTs) were identified considering the results described in Fig. 3e,f. The TcdA+/TcdB+/CDT+TPT was detected in 34.0% of the genomes (*n* = 18), which corresponded to all Clade-2 isolates, whereas the TcdA+/TcdB+/CDT− TPT was detected in 41.5% of the genomes (*n* = 22). An additional TPT (TcdA−/TcdB−/CDT−) was detected in the genomes of 24.5% (*n* = 13) of isolates that had lost the coding regions for the main toxins (Fig. 3g). The combination of TPTs shown by STs found as part of coexistence event 3 (Fig. 3c) completes the toxigenic arsenal in the same patient at the IH level (Fig. 3h). In general, the isolates with the TcdA+/TcdB+/CDT− TPT (Clade-1 members), showed the highest cytopathic effect (Fig. 3i).

The exhaustive search of toxin coding genes led to the identification of two accessory genes additional to *tcdR*, *tcdE*, and *tcdC*, corresponding to hypothetical proteins, which have already been described within *PaLoc*, that were restricted to most genomes in Clade-1 (marked with diamonds next to *tcdC* in Fig. 3e). For *CdtLoc*, although the mapping revealed the presence of coding regions for the two binary toxin subunits, they represented less than 50% of the total length of the genes (Supplementary Fig. S3), so the encoded proteins may not be functional and were considered negative for these regions during the analysis.

Atypical organizations in two *PaLoc* regions were also detected. One corresponded to an increase in the number of copies of the coding region for the holine-like protein that was identified in isolates obtained from 11 samples; however, this open reading frame was found in a single copy through the genomes (Supplementary Fig. S4a). In the four isolates established from sample 205 (obtained at IH level), although the majority of the *PaLoc* region was absent and they were classified as Tcd−/TcdB−/CDT− TPT contained an increased depth in holine-like protein region with respect to the average depth obtained in other *PaLoc* coding regions. The organization of this region in isolates obtained from sample 205 is like the organization in the sample 172 that was used as a model for the description of this organization (Supplementary Fig. S5a). The other atypical organization corresponded to loss of reads in a region close to *tcdA* in some isolates (marked with a red diamond in Fig. 3b). This represents a deletion towards the 3′ end of the coding region for *tcdA* for which the extended representation is shown in Supplementary Fig. S4b, which was present in Clade-2 isolates (Supplementary Fig. S5b) and affected the detection of this region during preliminary searches of the databases (Supplementary Fig. S2).

### High correlation between antimicrobial resistance molecular markers and *in vitro* MIC

**Figure 4:**
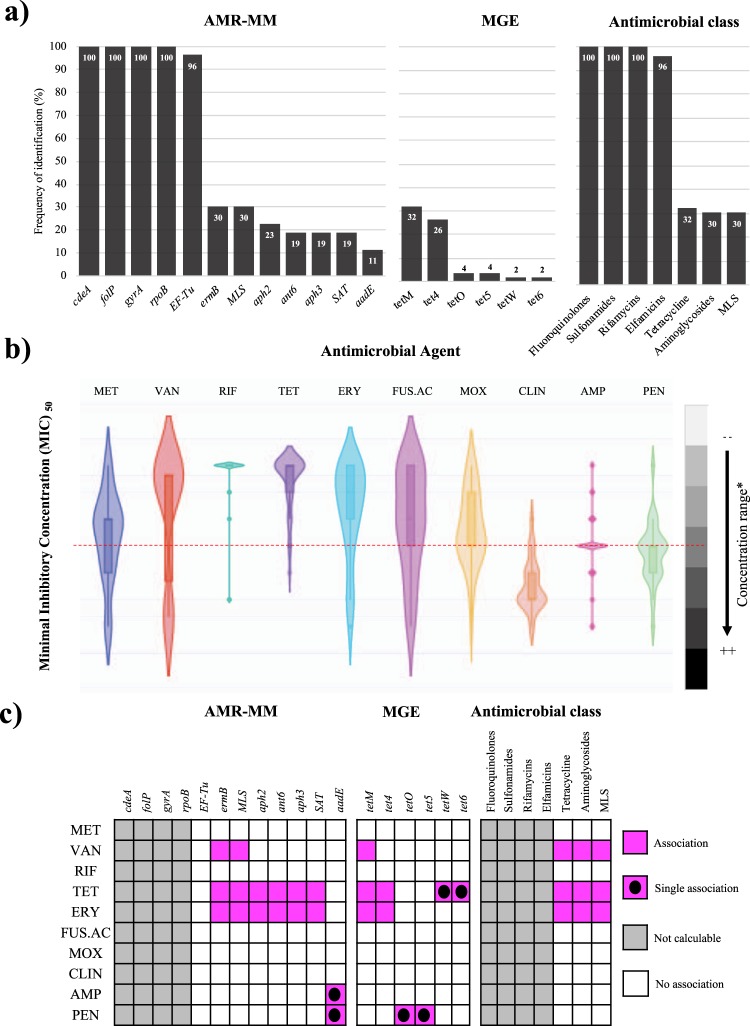
Genomic and phenotypic characterization of the antimicrobial resistance capacity of Colombian *Clostridium difficile* clinical isolates. (**a**) Frequency of antimicrobial resistance molecular markers (AMR-MMs) identification in reads obtained from whole genome sequencing of the clinical isolates. (**b**) MIC_50_ against 10 antimicrobial agents MET, metronidazole; VAN, vancomycin; TET, tetracycline; ERY, erythromycin; RIF, rifampicin; AMP, ampicillin; PEN, penicillin; FUS.AC, fusidic acid; CLI, clindamycin; MOX, moxifloxacin. The red dotted line represents the average concentration evaluated for each antimicrobial agent. MLS: Macrolide-lincosamide-streptogramin. The graphical representation of results was developed in Plotly server^66^. (**c**) Comparison of the detection frequency of the AMR-MMs with the MIC_50_ results. We identified as an association those comparisons that had a *p* < 0.05 after a Chi2 test. *Concentration range for MET, RIF and MOX was from 0.39 to 13; for AC.FUS, AMP and PEN was from 0.78 to 25; for VAN and TET was from 4.69 to 150; for ERY was from 1.17 to 38; and for CLI was from 0.15 to 5.

The existence of antimicrobial resistance molecular markers (AMR-MMs) was evaluated from whole genome sequences throughout comparisons against sequences deposited in eight data bases using Ariba software^13^. The results showed that the following four AMR-MMs were present in all isolates: *cdeA* and *gyrA* (associated with fluoroquinolones resistance), *folP* (that confers sulfonamides resistance) and *rpoB* (associated with rifamycins resistance). The following AMR-MMs in order were *EF-Tu* (associated with elfamycines resistance), present in 96% (*n* = 51) of the genomes. The identification of all other AMR-MMs was below 30% (*n* = 16), as in the case of *ermB* (conferring erythromycin resistance) and MLS (associated with macrolide, lincosamide and streptogramin resistance) (Fig. 4a). Moreover, less than 4% of the isolates contained markers associated with Mobile Genetic Elements (MGE): tetO, tet5, tetW, and tet6 (associated with tetracyclines resistance) (Fig. 4a). All analyzed genomes had molecular makers that have been previously associated to Fluoroquinolones, Sulfonamides and Rifamycins antimicrobial classes (Fig. 4a). The STs with higher number of AMR-MMs belong to the IH population (Supplementary Fig. S6), particularly those belonging to the Clade-2 with a total of 28–34 markers identified per genome (Supplementary Table 3).

The MIC_50_ for 10 antimicrobials was determined for *C*. *difficile* (metronidazole, vancomycin, tetracycline, erythromycin, rifampicin, ampicillin, penicillin, fusidic acid, clindamycin, and moxifloxacin), with the aim to identify the real effect of AMR-MMs in phenotypic resistance. We found low sensitivity of the isolates to metronidazole, one of the most commonly used agents for the treatment of CDI, but adequate sensitivity for vancomycin and rifampicin, which are also recommended for its therapeutic management. Clindamycin, ampicillin, and penicillin showed limited ability to inhibit colony proliferation, as confirmed by both the MIC_50_ (Fig. 4b) and MIC_90_ (Supplementary Fig. S7). Tetracycline, erythromycin and vancomycin were the antimicrobial agents with higher number of associations with AMR-MMs presence. Conversely, a lack of association was evident for metronidazole, rifampicin, fusidic acid, moxifloxacin and clindamycin (Fig. 4c).

### Colombian isolates show increased sporulation capacity and number of viable spores

**Figure 5:**
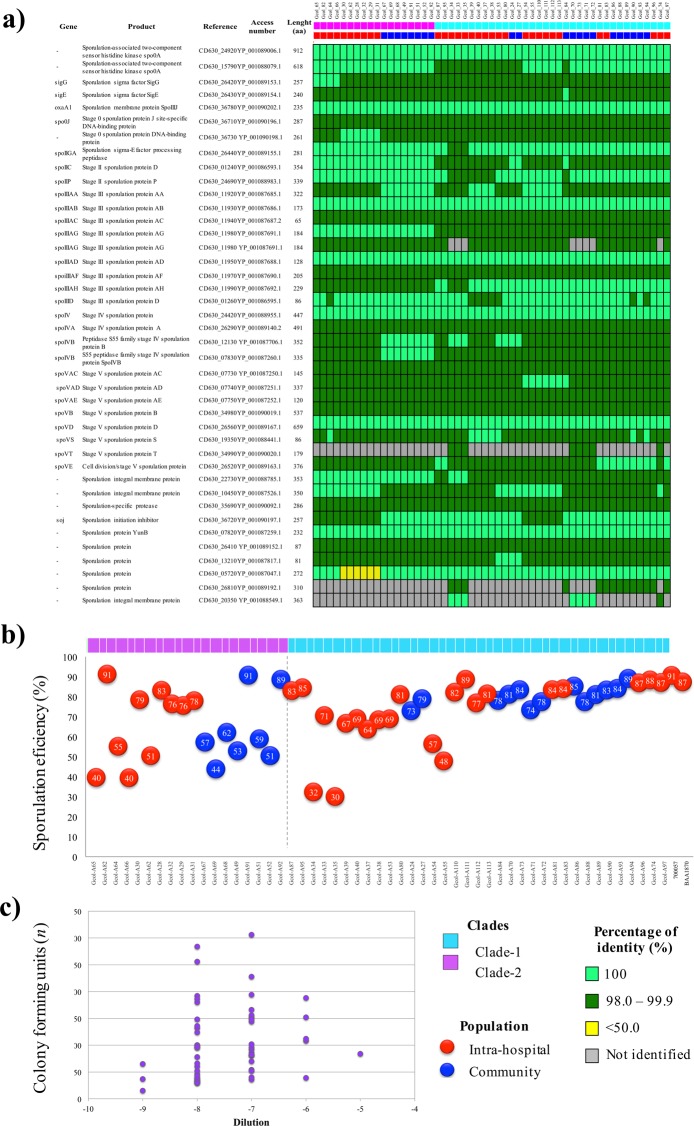
Genomic and phenotypic characterization of spore producing capacity and new CFU production. (**a**) Proteins involved in the sporulation process detected by comparisons against the sequences of the proteins involved in these processes, broadly characterized in the *C*. *difficile* 630 reference strain. (**b**) Sporulation efficiency detected after *in vitro* culture induction over 70:30 medium. (**c**) Count of CFUs detected by dilution after spore purification and subsequent growth induction over taurocholate supplemented medium.

The presence of proteins involved in the sporulation/germination process was evaluated initially by comparing their predicted amino acid sequences (Fig. 5a). We found that the Colombian CDs produced at least 38 of the 47 proteins involved in sporulation processes reported in ClosIndb for the reference strain *C*. *difficile* 630 uid57679^14^. Most of these proteins shared ≥98.0% identity; the exception was the reference sporulation protein CD630_05720, which shared <50.0% identity with the proteins in five of the Clade-2 isolates. We also found a double copy of the stage III sporulation protein AG (CD630_11980) in most of the isolates, except eight members of Clade-1. Three proteins encoded by a limited number of genomes (8–13) were also found, namely, CD630_34990, CD630_26810, and CD630_20350.

The phenotypic evaluation of the sporulation efficiency was higher for the Clade-1 than for the Clade-2 isolates (Fig. 5b), except for Gcol_A34, Gcol_A35, Gcol_A54, and Gcol_A55 for which the efficiency was <60%. The results were more heterogeneous for isolates in Clade-2, where the sporulation efficiency ranged from 40–91%. No differences were found by population. In the case of multiple infection events, the sporulation efficiencies of all the isolates were >80%.

The spores were purified and used to evaluate the capacity of the isolates to generate new CFUs, as an indicator of germination efficiency (Fig. 5c). The dilution at which the isolates generated colonies in the range 30–300 CFUs was then determined. It was found that the isolates generated CFUs in dilutions from 10^−6^ to 10^−9^, whereas the ATCC® control strains 700057™ and BAA-1870™ generated CFUs in dilutions from 10^−5^ and 10^−6^, respectively, which indicated the higher germination capacity of Colombian *C*. *difficile* isolates than the control strain.

## Discussion

Genomic epidemiology is one of the most useful strategies to detect, characterize, and monitor pathogens that have an impact on human health^15^. Although a large number of studies have elucidated the genomic epidemiology of *C*. *difficile*^16,17^, only a limited number of studies have focused on Latin America. One such study of *C*. *difficile* in Costa Rica not only identified hypervirulent CD strains at the local level, but also described profiles of toxin production that differed from those reported previously, and was the first study to reveal the high genomic diversity of *C*. *difficile* in this region of the world^18^. The absence of a routine *C*. *difficile* diagnosis strategy in Colombia led our group to investigate toxigenic profiles in patients with diarrhea. We detected circulating hypervirulent *C*. *difficile* strains and revealed the importance of the CO population in the epidemiology of *C*. *difficile* in Colombia. We also found the coexistence of *C*. *difficile* either with different STs or with the same ST but different toxins cassettes^9,19^. Considering the evidence of the high diversity of circulating *C*. *difficile* in Colombia, we performed genomic and phenotypic characterization of the *C*. *difficile* strains circulating in Bogotá (Fig. 1).

During the first phase of analysis, the typing strategy traditionally accepted for *C*. *difficile* based on MLST^12^ was applied to the complete genomes of the isolates, which showed that most of the circulating STs in Colombian *C*. *difficile* strains belong to Clade-1, with 9 STs (Fig. 1A,B). This agrees with the available data about the molecular epidemiology of this pathogen at a global level, which describes Clade-1 as the most heterogeneous clade because it contains STs with high frequency of detection in different regions of the world^20^. The rest of the isolates belonged to Clade-2, with only 2 STs corresponding to ST-41 and ST-1 (Fig. 1A,B), a clade associated with hypervirulent strains that lead to a severe clinical picture and high recurrence events^21^. ST-8 had the highest frequency of detection among the isolates (Fig. 1B) and was the only ST present in the two populations evaluated (IH/CO, Fig. 1C). ST-41 was the most frequently found ST at the IH level (Fig. 1C) and it has been associated with severe inflammatory disease and disruption of the intestinal mucosa^22^. ST-1 was the most frequently found ST in the CO population and it was present only in this population (Fig. 1C). These results are of particular importance because ST-1 (Clade-2) was associated with hypervirulent strains involved in outbreaks in North America and Europe, which is why they are recognized as an important public health problem^21,23,24^. The MLST analysis also allowed the identification of three coexistence events of two STs simultaneously in the same patient (Fig. 1A), being the coexistence event 3 caused by Clade-1 and Clade-2 STs. Two of these events were identified from CO samples (events 2 and 3) and ST-8 was the only ST that was present consistently in all the events, which could be due to its widely reported frequency^25^. Although in Colombia typing schemes have been applied to a small number of isolates of *C*. *difficile*, these studies have been based on ribotyping and had a purely descriptive approach^26^. The comparison of the findings with studies worldwide reveals a similar profile in that ST-8, the most frequent in this study, has been reported in the United Kingdom^27^, Australia^28^, China^29^ and United States^30^, usually associated with recurrent infections, in coinfection with a second ST, also showing substitutions associated with resistance to antibiotics (at the level of *gyrA* and *gyrB*)^27,29^.

Herein, we generated a tree based on a core genome (Fig. 2a). Isolate clustering with members of the two main *C*. *difficile* clades (Clade-1 and Clade-2), and the identification of C5 as a possible common ancestor, is consistent with previous reports^20^. These results are aligned with recent reports in which a core genome MLST (cgMLST) scheme was proposed as a useful tool to describe the population structure of *C*. *difficile* at the genetic level. The high percentage of genes (71.4%) that were part of the accessory genome and the inconsistencies in the topology of the phylogenetic reconstruction of the concatenated sequences of the accessory genes (Fig. 2c), which included a change in the relationship between some STs, as well as the high number of accessory and unique genes (Fig. 2d–f) confirmed the dynamic character of the *C*. *difficile* genome.

Comparisons of the genome sequences of the established isolates with the information available in databases revealed the high frequency with which the isolates obtained at the IH and CO levels transport toxin coding genes (Supplementary Fig. S2). However, the unexpected findings in Clade-2 and the cytotoxic capacity detected after phenotypic tests, revealed that this strategy had limitations in accurately describing the organization of circulating *C*. *difficile* isolates in Colombia. The detection of *toxB*, which leads the pathogenic effect on target cells, was consistent^31^, which indicated its importance for *C*. *difficile* colonization^32^. The changes in the organization of *PaLoc* regions that we detected (Fig. 3) corresponded to gain of *tcdE*, which encodes the holine-like protein in Clade-1 genomes initially considered non-toxigenic (Supplementary Fig. S2), and the loss of a region of *tcdA* in Clade-2 genomes, which could affect its detection, indicated that the highly diverse strains circulating in Latin America could escape the diagnostic strategies traditionally used for CD. Our study has revealed atypical organizations in Clade-1 and Clade-2, which suggests the urgent need to develop novel diagnostic strategies for *C*. *difficile* in Latin America.

The significantly greater cytopathic effect shown by the TcdA+/TcdB+/CDT− TPT compared with TcdA+/TcdB+/CDT+ TPT for all the main toxins (Fig. 3i), could be explained either by greater efficiency of the action of the proteins encoded in *PaLoc* or by the effect of additional genes that have not yet been analyzed. In *PaLoc*, the two additional accessory genes to those traditionally reported (*tcdR*, *tcdE*, and *tcdC*) could be involved, although they are currently only recognized as coding hypothetical proteins. They could play a role during the secretion/action process of TcdA and/or TcdB; however, these two additional accessory genes need to be characterized in order to identify their potential role during the *C*. *difficile* infection process.

Four AMR-MMs were detected in all the evaluated genomes (Fig. 4a). Two of these conferring resistance to fluoroquinolones (*cdeA*^33^ and *gyrA*^34^), while the other two have been associated with sulfonamides (*folP*^35^) and rifamycin (*rpoB*^36^) resistance. Three of these highly prevalent markers correspond to mutations in constitutive genes (*gyrA*, *folP* and *rpoB*), it confirms the ability of *C*. *difficile* to modify even constitutive proteins as part of the process of adaptation to hostile environments such as the human intestine, particularly when it has been exposed to antibiotic therapy^37^. The existence of these four AMR-MMs in all the characterized genomes indicate that this type of modification could be setting as part of the core *C*. *difficile* genome, although it has been historically proposed that they may have been transferred through mobile genetic elements, mainly plasmids^38^. Additionally, besides being the clade with the most genes coding for toxins (Fig. 3e), Clade-2 had isolates with the highest number of AMR-MMs (Fig. 4a), and this clade also showed reduced susceptibility to a higher number of antimicrobials than Clade-1 isolates. Interestingly, the analyzed isolates obtained from IH environment showed higher number of AMR-MMs than those established from CO population (Supplementary Fig. S6). Therefore, these findings could be because of *C*. *difficile* adaptation due to exposure to antimicrobial agents that led to selective pressure^39^, but the history of antibiotic consumption of the individual patients who provided the samples in this study is unknown and is a limitation of this study.

The identification of at least 38 of the proteins involved in the different phases of sporulation by comparison with the *C*. *difficile* 630 reference strain revealed that, in most cases, they shared >98.0% identities (Fig. 5a)^40^. Some specific exceptions were found, such as the absence of the gene encoding the integral membrane protein CD630_20350, which was found in only eight of the genomes. This protein has been reported as not strictly required for the sporulation or resistance of spores^40^; therefore, it may be that the absence of genes occurs when the encoded protein is not essential for sporulation. Interestingly, we identified the stage III sporulation protein AG (CD630_11980), which had two copies in most of the genomes (45 out of 53). This protein, which is encoded in the spoIIIAABCDEFGH operon, under the control of the sigma G factor and expressed during phase 3 of sporulation, has been reported as strictly conserved among species in phylum Firmicutes that form spores (http://www.ebi.ac.uk/interpro/entry/IPR014195).

When analyzing the sporulation percentage determined during the phenotypic tests (Fig. 5b), we found that, although the results were heterogeneous for Clade-2 isolates, most of the Clade-1 isolates had sporulation percentages >60%, the exception were Gcol.A33, Gcol.A34, Gcol.A54, and Gcol.A55. The genomes of two of these isolates had only a single copy of CD630_11980 and the isolates had the lowest percentage of sporulation (≤32%).

In general, the confirmation of the plasticity of the *C*. *difficile* genome, the presence of STs with more than one toxigenic profile, the identification of infection events by more than one ST, and the variations found in cytotoxic capacity, sporulation, germination, and profiles of resistance to antibiotics, represent factors at the genomic and phenotypic levels that contribute to the knowledge of circulating *C*. *difficile* characteristics in Colombia. This study presents related limitations with the small sample size and reduced number of isolates, that limit the extrapolation of the data, therefore, future studies should include a larger sample size in order to support the results obtained here. However, our results provide evidence of the microdiversity that usually defines *C*. *difficile* populations and support the hypothesis that this opportunistic pathogen is maintained in continuous evolution processes^41^ that impact on its adaptation during persistent infection processes^42^. Additionally, the differential presence of different groups of genes and their correlation with the phenotypic profiles described here could have a profound impact on *C*. *difficile* ecology because horizontal gene transfer may favor acquisition of virulence and the subsequent transition from a microorganism environmental lifestyle to a pathogen, as was proposed in the hypothesis of virulence adaptive polymorphisms, which has been tested in *Vibrio cholerae*^43^. This represents the first genomic and phenotypic approach conducted in Colombia and in Latin-America to our knowledge. Further studies in the region are needed to obtain the broad genomic epidemiology of CD.

## Materials and Methods

### Ethics approval and consent to participate

The Universidad del Rosario’s Research Ethics Committee approved the initial study aimed to detect and isolate CDI in fecal samples from patients with diarrhea in Bogotá, Colombia, through the act No. 290, July 27, 2015. All patients included in this study agreed to participate and signed informed consent forms agreeing to their participation in the study. All methods were performed in accordance with the Helsinki declaration and the Colombian ministry of health and social protection guidelines as approved by the certificate of the Universidad del Rosario’s Research Ethics Committee. In the context of this study, no clinical or other metadata were analyzed about the patients.

### Clinical isolates

Fifty-three isolates were established from stool samples of patients with diarrhea who attended two healthcare centers in Bogotá, Colombia (Hospital Universitario Mayor – Méderi and Fundación Clínica Shaio). The methodology for the collection of the samples, CDI detection and establishment of isolates was previously reported by our group^9^. Briefly, an aliquot of the stool sample collected from each patient was spread on ChromID C. difficile CDIF (bioMérieux) and incubated for 48 hours at 37 °C under anaerobic conditions, using the GasPak EZ Anaerobe Pouch system (Becton Dickinson)^44^. The colonies with the macroscopic morphology described by the manufacturer were extended on Trypticase ™ Soy Agar (TSA) with 5% Sheep Blood (Becton Dickinson), and subsequently incubated under the aforementioned conditions. A verification by microscopic inspection by routine interpretation by Gram stain was performed. The cellular biomass of the verified colonies was extended by massive seeding on medium TSA, which was later recovered for the isolate’s establishment. Multiple colonies were recovered for each sample because of the possible coexistence of *C*. *difficile* genotypes^10^.

The isolates were assigned to community ‘CO’ population when the patients with diarrhea attended the emergency service of the participating health-care centers and their time since admission to the medical center did not exceed 48 hours, or to intra-hospital ‘IH’ population, when the patient took three days or more to stay in the different services within the participating health centers (Supplementary Table S1), as was previously described^45^.

### DNA extraction and whole genome sequencing (WGS)

DNA was extracted using an Ultraclean Blood Spin DNA Isolation kit (MoBio Laboratories, Carlsbad, CA, USA) following the manufacturer’s instructions. The WGS of the selected isolates was carried out by Novogene Bioinformatics Technology Co., Ltd. (Beijing, China) using the Illumina HiSeq X-TEN platform. The quality control, assembly and genome identification can be retrieved in Supplementary Text S1.

### Annotation and comparative genomics analysis

An automated annotation strategy was applied to the multi-FASTA files obtained from the assembly process. This strategy uses PROKKA v1.11^46^ and is complemented by an improvement by comparison against genus-specific databases in RefSeq.^47^, which was applied to all the genomes included in the analyzed data set. First, circular visualization of the genomes was carried out in the CGview server^48^, then pairwise comparisons were made to identify differences between the genomes using the tool based on the BLAST algorithm that is included in the CGview server. The GFF files obtained from the annotation process were used to determine the pangenome using the Roary tool^49^, with a percentage of identity of 95% and definition of the core genome of 99%. A presence/absence matrix of the genes that were part of the core or accessory genome was constructed as a basis to graphically represent the pangenome results using the Python script roary_plots.py^50^.

### Phylogenetic analysis

The concatenated sequences of the seven housekeeping genes that are part of the MLST scheme were extracted and aligned using the script align_seqs.py, considering the MUSCLE method^51^. For phylogenetic reconstructions, maximum likelihood trees were built from the alignments of both the MLST scheme and the core genome using FastTree Version 2.1.9 with double precision^52^. The robustness of the nodes was evaluated using the bootstrap method with 1,000 replicates^53^. The phylogenetic trees were visualized in the web tool Interactive Tree Of Life V3 (http://itol.embl.de)^54^.

The sequences of 15 high-quality reference genomes provided by the Wellcome Trust Sanger Institute (https://www.sanger.ac.uk/science/data/reference-genomes-clostridium-difficile) were included in the comparative analysis. These genomes belong to different *C*. *difficile* ribotypes (including those associated with the hypervirulent strains RT027 and RT078) and were considered as representative of the clades currently accepted for intra-taxa classification of CD^20^. Detailed information for the reference genomes is described in Supplementary Table S4. A multi-FASTA alignment file and the SNP-sites program to detect and extract SNPs were used to analyze the informative sites from the phylogenetic approach^55^. A pairwise SNP distance matrix from a FASTA alignment of the core genomes was generated to compare the 68 individual isolates included in the pangenome analysis. In addition, a detailed analysis of the accessory genome was conducted through the generation of a phylogenetic reconstruction from the binary gene presence matrix and the subsequent evaluation of the total number of accessory and unique genes by isolate.

### Analysis of coding sequences for the main toxins

The presence of query coding sequences was evaluated using a pipeline composed by an initial step of mapping against reference sequences based on Burrows-Wheeler Aligner (BWA)^56^, using BWA-MEM high-performance algorithm for Illumina reads^57^. Secondly, the conversion, ordering, and indexing of the reads were carried out in the Sequence Alignment/Map (SAM) tools^58^. The pipeline was completed with a quality statistics calculation step of aligned sequences using flagstat and stats in SAMtools^58^. The genomes were visualized in Artemis tool^59^.

### Identification of virulence factors from WGS

The identification of virulence factors in the complete genomes of the isolates was directed to three main aspects: toxin coding genes, other virulence factors, and antimicrobial resistance molecular markers (AMR-MM)^8^, using Ariba^13^ (Supplementary Fig. S1a). Comparisons with the reports available in the eight databases available in this software (card^60^, vfdb_core^61^, arganot^62^, megares^63^, plasmidfinder^64^, resfinder^65^, srst2_arganot^62^ and virulencefinder^65^) were done. The graphical representation of results was developed in Plotly server^66^. The presence of toxin coding genes was confirmed by mapping them against reference sequences of *PaLoc* and *CdtLoc*. CD-HIT software was used to cluster sequences very similar to the proteins involved in the sporulation/germination processes^67^. This analysis involved the definition of a set of reference proteins known to be involved in sporulation pathways in the *C*. *difficile* 630 uid57679 reference strain. The amino acid sequences of those reference proteins were exported from ClosIndb database, a data repository for analysis of *Clostridium* species^14^. An identity percentage ≥40.0% and a K-mer = 2 (defined as a subsequence of length K = 2, used to index the amino acid sequences) were the parameters considered for cluster definition.

### Phenotypic characterization

The cryopreserved isolates were activated and used to develop: (i) cytotoxicity tests^68^; (ii) Minimal inhibitory concentration (MIC) of 10 antimicrobial agents (metronidazole, vancomycin, rifampicin, tetracycline, erythromycin, fusidic acid, moxifloxacin, clindamycin, ampicillin, and penicillin; (Supplementary Table S5)) 50 (MIC_50_) and 90 (MIC_90_) determination^69^, and (iii) sporulation efficiency and number of viable spores^70^. A graphical description of complete methodology used to phenotypically characterize the isolates studied is described in the Supplementary Fig. S1b. Chi2 tests were developed to evaluate the existence of associations between the presence/absence of AMR-MMs and the categories established for MIC_50_ results.

## Supplementary information

Supplementary information

## Data Availability

The set of genomes analyzed in this study were deposited at DDBJ/ENA/GenBank as part of the BioProject PRJNA551724.
