# Supplementary material for: Integrated genomic epidemiology and phenotypic profiling of Clostridium difficile across intra-hospital and community populations in Colombia
Source: Sci Rep. 2019 Aug 5;9:11293. doi: 10.1038/s41598-019-47688-2 (PMC6683185; doi:10.1038/s41598-019-47688-2)
Supplement: Supplementary file 1 — Supplementary information [file 41598_2019_47688_MOESM1_ESM.pdf]

Integrated genomic epidemiology and phenotypic profiling of *Clostridium difficile* across intra-hospital and community populations in Colombia

Marina Muñoz[a,b], Daniel Restrepo-Montoya[a,c], Nitin Kumar[d], Gregorio Iraola[e,f], Milena Camargo[g,h], Diana Díaz-Arévalo[g,i,j], Nelly S. Roa-Molina[k], Mayra A. Tellez[k], Giovanny Herrera[a,l], Dora I. Ríos-Chaparro[a], Claudia Birchenall[m], Darío Pinilla[m], Juan M. Pardo-Oviedo[m], Giovanny Rodríguez-Leguizamón[m], Diego F. Josa[n], Trevor D. Lawley[d], Manuel A. Patarroyo[g,h] and Juan David Ramírez[a]*

[a] Grupo de Investigaciones Microbiológicas–UR (GIMUR), Programa de Biología, Facultad de Ciencias Naturales y Matemáticas, Universidad del Rosario, Bogotá, Colombia
[b] Posgrado Interfacultades Doctorado en Biotecnología, Facultad de Ciencias, Universidad Nacional de Colombia, Bogotá, Colombia
[c] Genomics and Bioinformatics Department, North Dakota State University, Fargo, North Dakota, USA
[d] Host-Microbiota Interactions Laboratory, Wellcome Sanger Institute, Hinxton, UK
[e] Microbial Genomics Laboratory, Institut Pasteur Montevideo, Montevideo, Uruguay
[f] Center for Integrative Biology, Universidad Mayor, Santiago de Chile, Chile
[g] Molecular Biology and Immunology Department, Fundación Instituto de Inmunología de Colombia (FIDIC), Bogotá, Colombia
[h] School of Medicine and Health Sciences, Universidad del Rosario, Bogotá, Colombia
[i] Faculty of Animal Sciences, Universidad de Ciencias Aplicadas y Ambientales (UDCA), Bogotá, Colombia
[j] Hygea group, Faculty of Health Sciences, Universidad de Boyacá, Tunja, Colombia
[k] Centro de Investigaciones Odontológicas, Facultad de Odontología, Pontificia Universidad Javeriana, Bogotá, Colombia
[l] PhD Programme in Biomedical and Biological Sciences, Faculty of Natural Sciences and Mathematics / School of Medicine and Health Sciences, Universidad del Rosario, Bogotá, Colombia
[m] Hospital Universitario Mayor – Méderi, Universidad del Rosario, Bogotá, Colombia
[n] Fundación Clínica Shaio, Bogotá, Colombia

# Corresponding author
Juan David Ramírez, juand.ramirez@urosario.edu.co

Supplementary information

Supplementary Text S1. Quality control, assembly and genome identification of analyzed genomes.

Quality control

The general scheme for the whole-genome analysis is shown in Additional file 1. First, the quality of the obtained reads was determined by comparing them with the CD reference strains available in PATRIC 3.5.4. [https://www.patricbrc.org/view/GenomeList/; Search criteria: Genomes/Clostridium])[1,2], European Nucleotide Archive[3] and National Center for Biotechnology Information databases. Identity was estimated against the most closely related reference genomes (i.e., those that contributed to the verification of the taxonomic assignment) and the depth of the sequencing, defined as the average number of reads per genome position, was determined. A complementary quality verification procedure was carried out using GenomeQC_Filter_v1-5 script, an in house Sanger Perl script which considers a maximum of 400 contigs per genome and maximum size of each genome of 8 Mbp, and extracts the 16S ribosomal RNA gene (rRNA-16S) sequence[4], for comparison using the alignment tool of the SILVA database[5].

Assembly and genome identification

The reads obtained from the sequencing process were assembled *de novo* to avoid possible biases, considering the highly dynamic nature of the CD genome. We used the improved protocol for Illumina data designed for prokaryotes[6], which generates multiple assemblies with Velvet v1.2[7] and VelvetOptimiser v2.2.5 (https://packages.debian.org/buster/velvetoptimiser), followed by an assembly improvement step considering the best N50. The best prediction was then assembled using SSPACE[8] and the spaces between contigs were filled using GapFiller[9].

The assembled genomes were subjected to a delimiting species step using average identity of nucleotides (ANI)[10]. This tool is available as the anib function in Taxxo v1.0, an R package[11,12]. ANI >95.0 was considered to confirm that the genomes belonged to the same species.

The sequence types (STs) circulating in Colombia were determined by multilocus *in silico* typing using MLSTcheck[13], which makes comparisons with the PubMLST database (https://pubmlst.org/cdifficile/)[14] that allow the identification of the seven constitutive gene sequences that are part of the standardized scheme for intra-taxa typing of CD[15] and subsequent assignment of the allelic profiles.

Supplementary Fig. S1. Schematic representation of the methodology used to characterize the 53 CD Colombian clinical isolates used in this study. a) Whole-genome analysis of the sequences obtained from the isolates. b) Phenotypic characterization of the isolates. In b panel the cytotoxicity assays are remark in pink color, the minimum inhibitory concentration tests in purple color and the sporulation efficiency/viable spores in green color. SBHI, supplemented brain heart infusion (BHI); UFC, unit-forming colony.

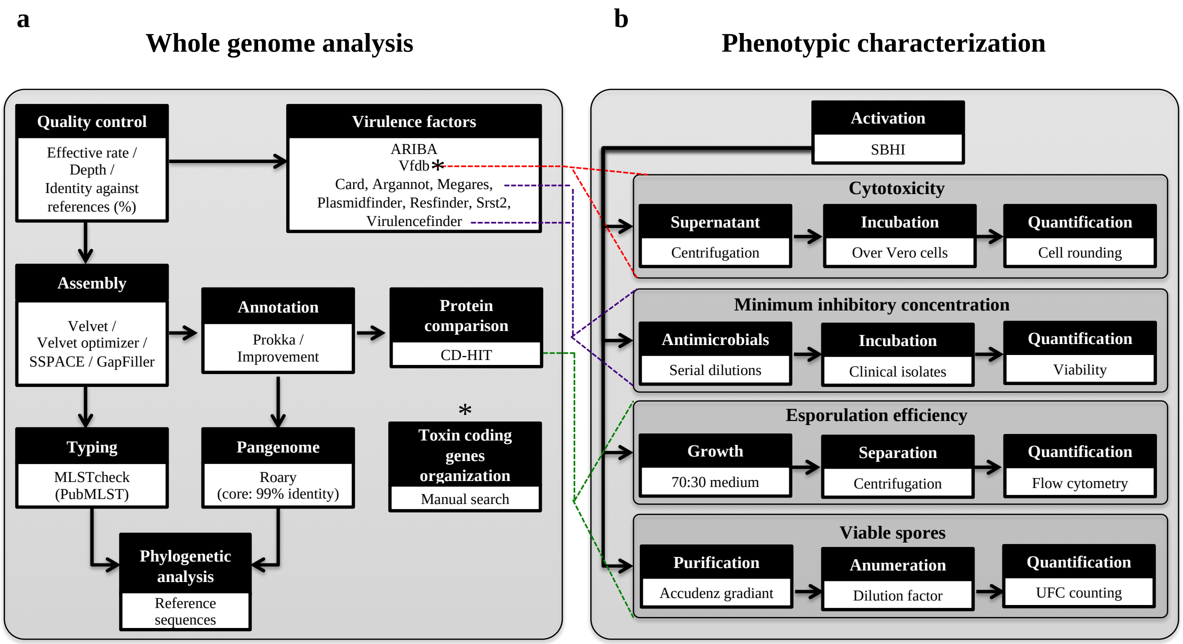

Supplementary Fig. S2. Sequences associated with the main CD toxin coding genes identified using ARIBA.

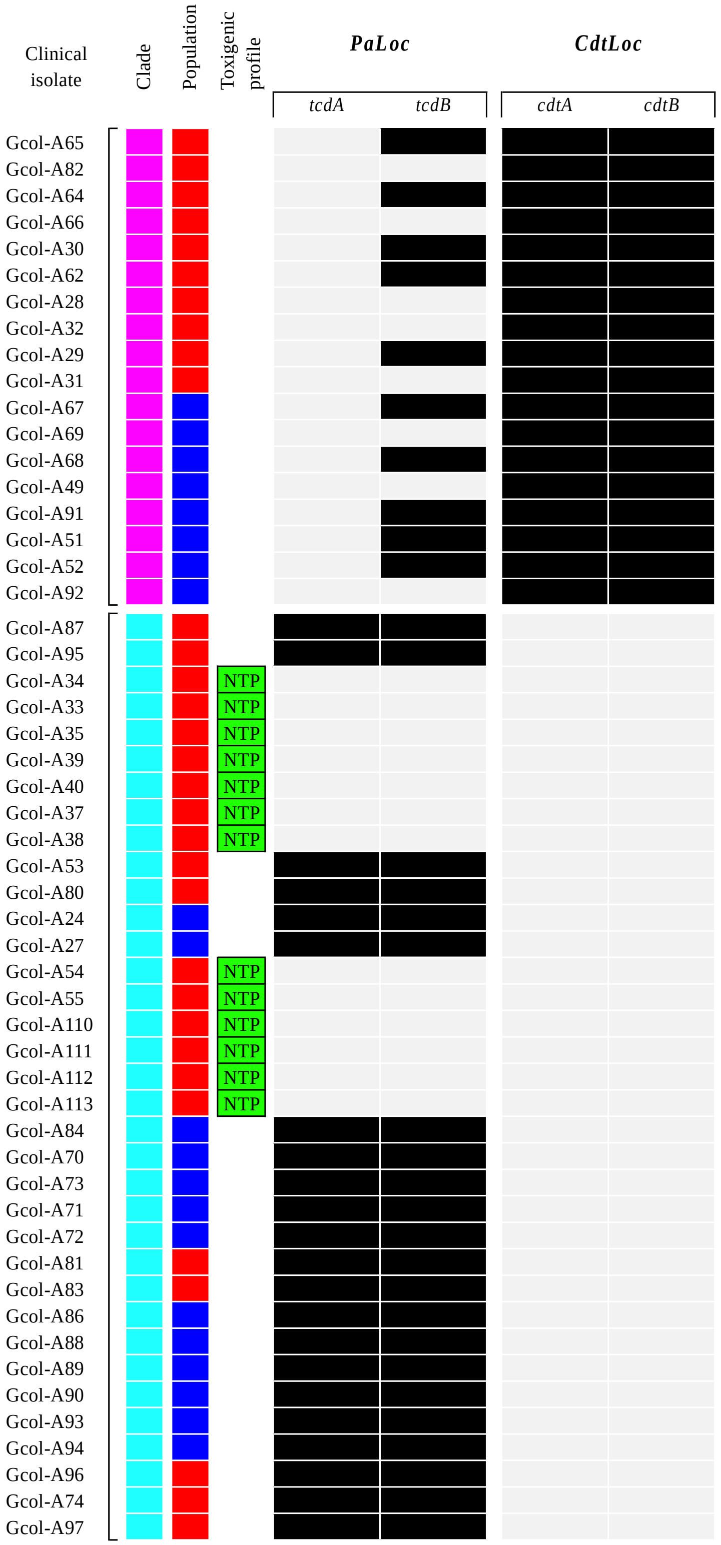

Supplementary Fig. S3. *CdtLoc* mapping results. Multiple alignment of consensus sequence resulting of mapping for each *C. difficile* short reads files against the reference sequence YP_001087137.

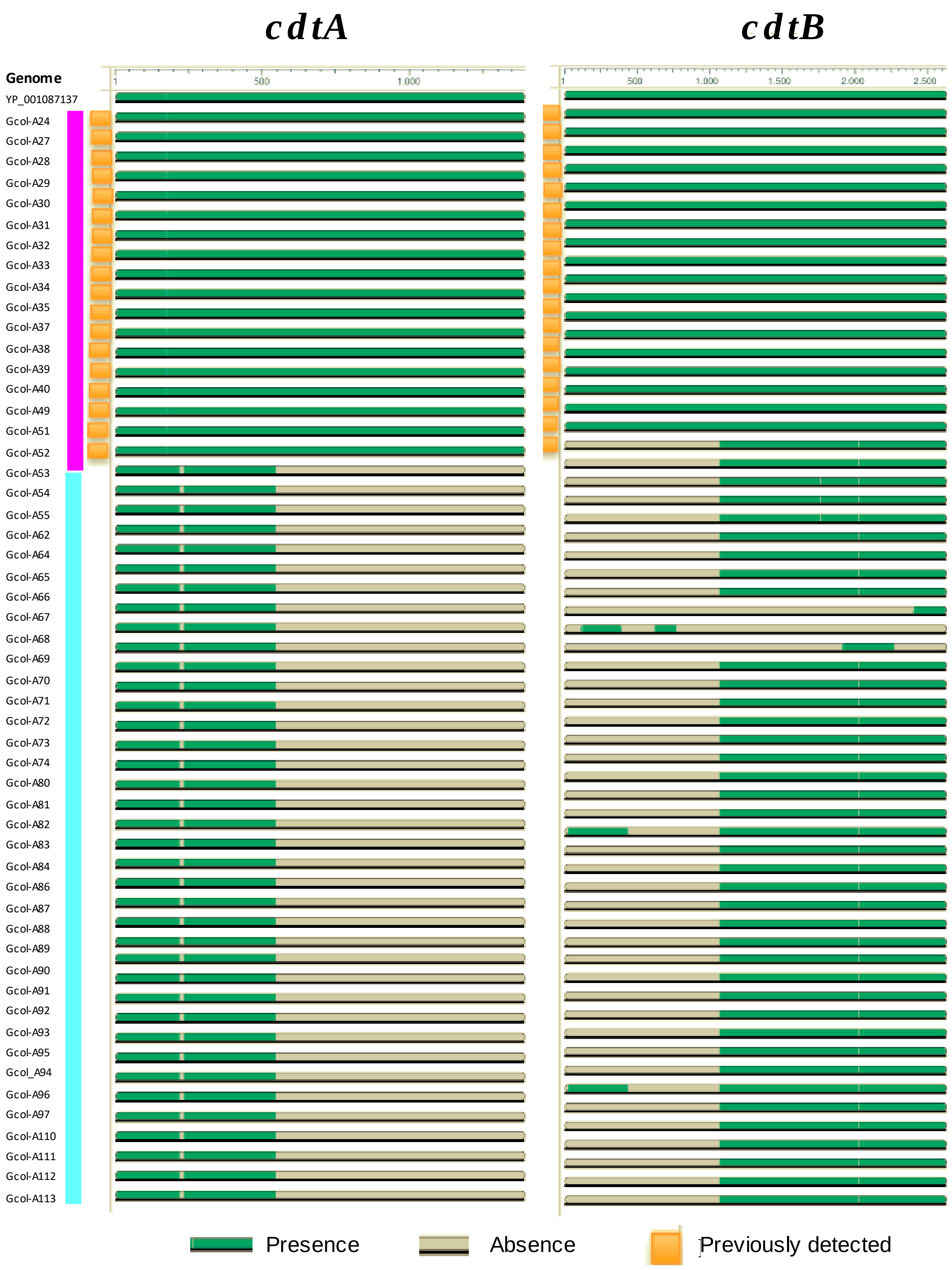

Supplementary Fig. S4. Changes in depth mapping against reference sequence YP_001087137 with respect to the average depth obtained in *PaLoc* codifying region. a) increase in depth in holine-like protein coding region. b) lack of mapping in 3'region of *tcdA* open reading frame.

a)

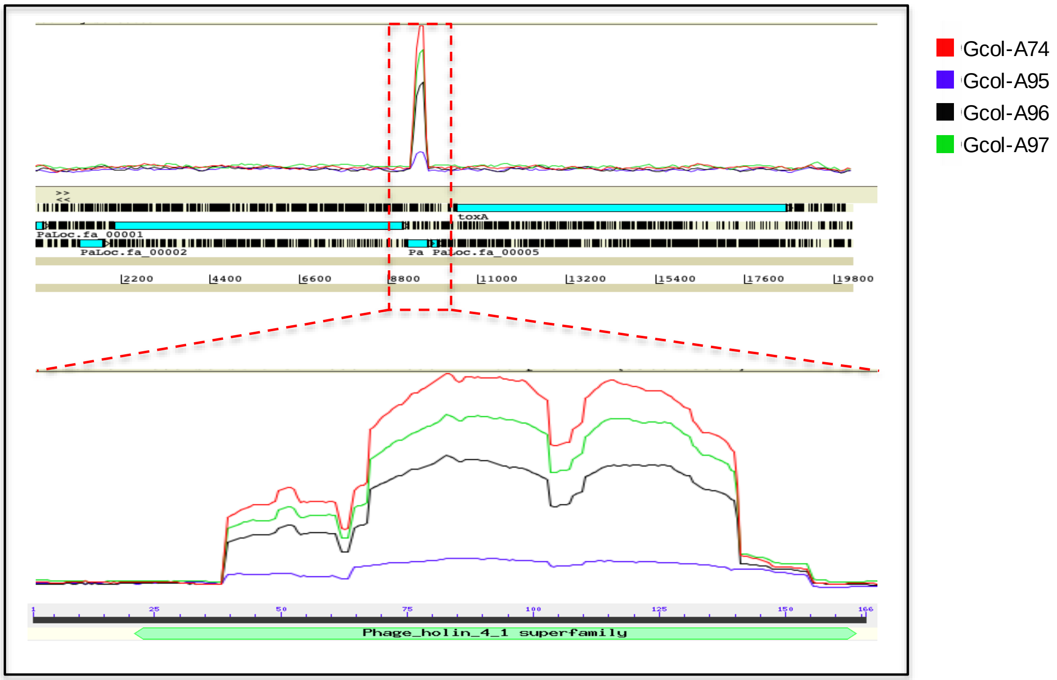

b)

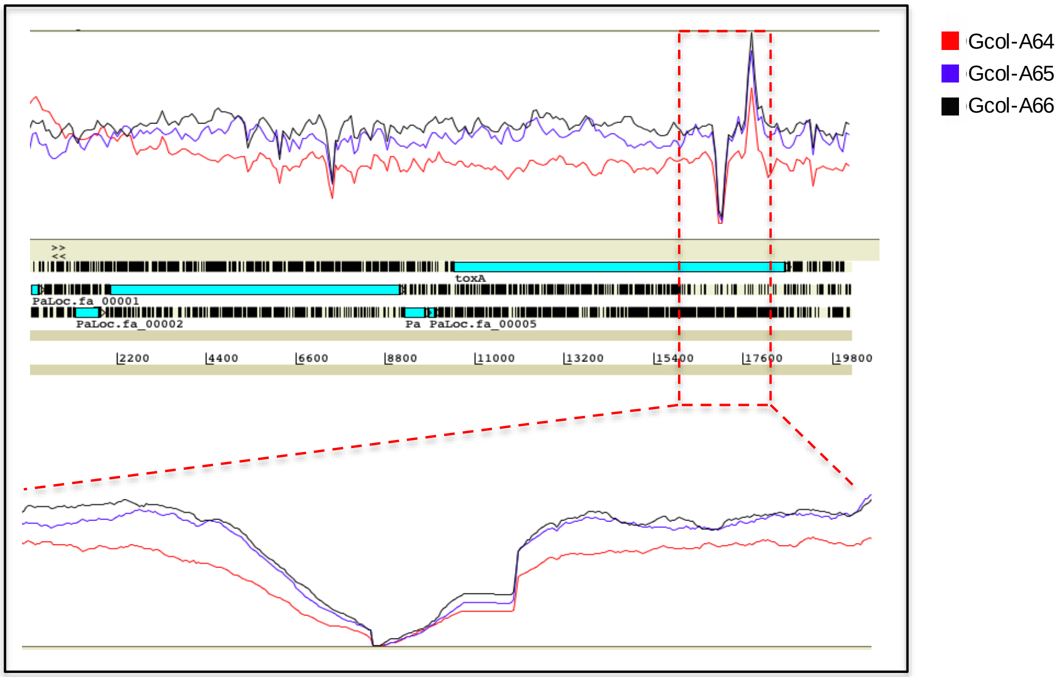

Supplementary Fig. S5. Atypical organizations in two *PaLoc* regions.

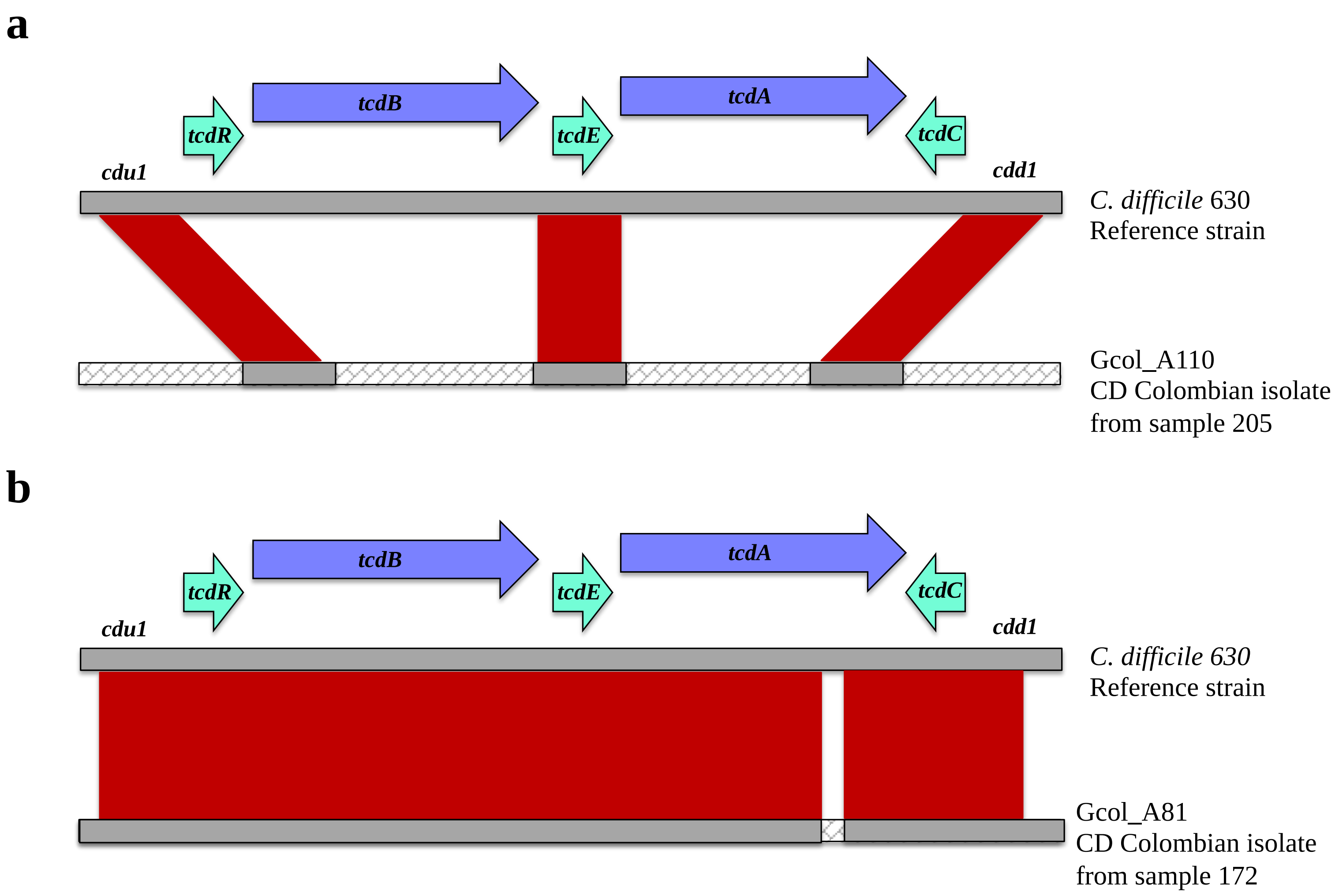

Supplementary Fig. S6. Antimicrobial molecular markers (AMR-MMs) identified per genome using Ariba tool[16]. The black and dotted boxes indicate the genomes of IH population with a greater number of AMR-MMs

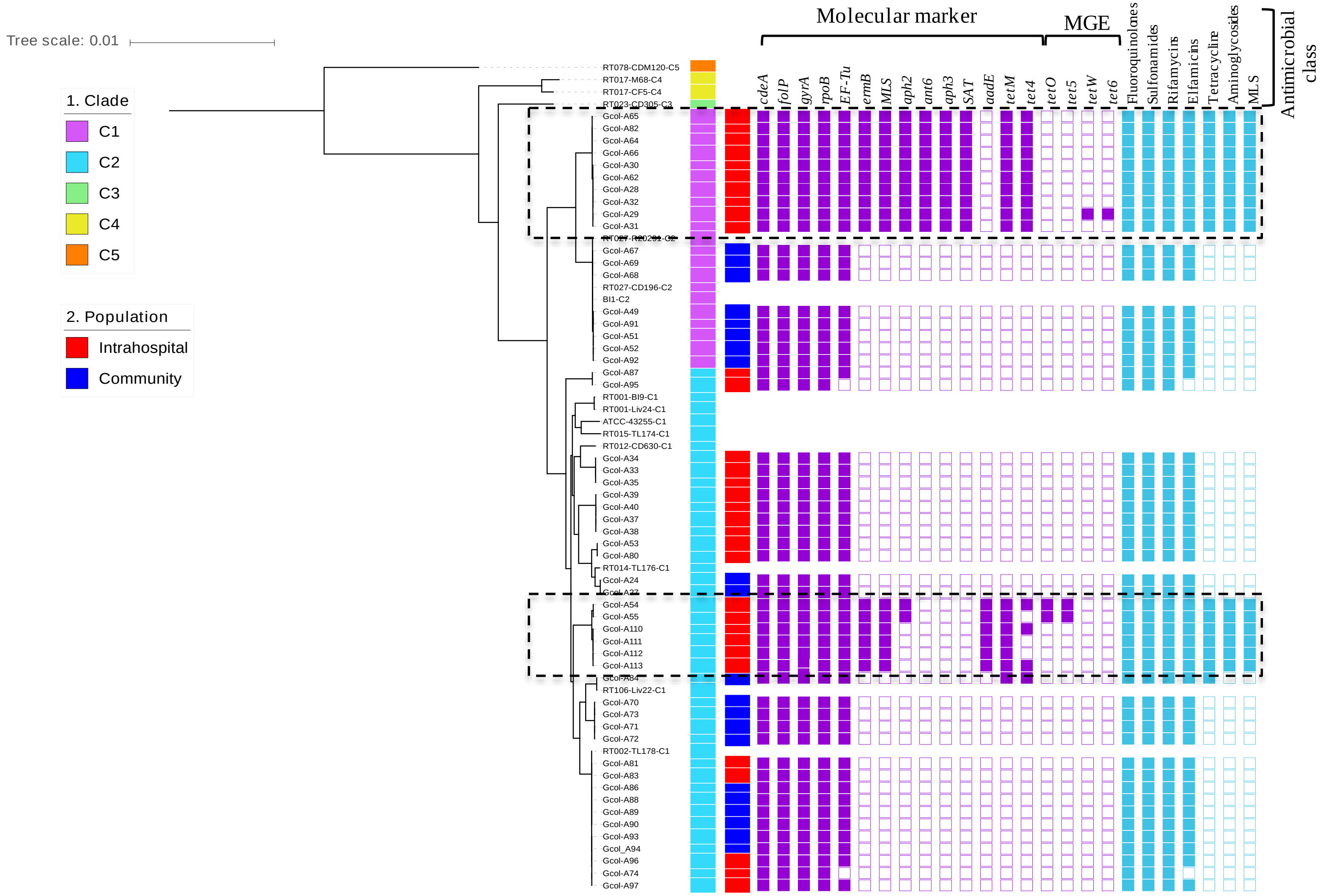

Supplementary Fig. S7. Minimal Inhibitory Concentration 90 (MIC_{90}).

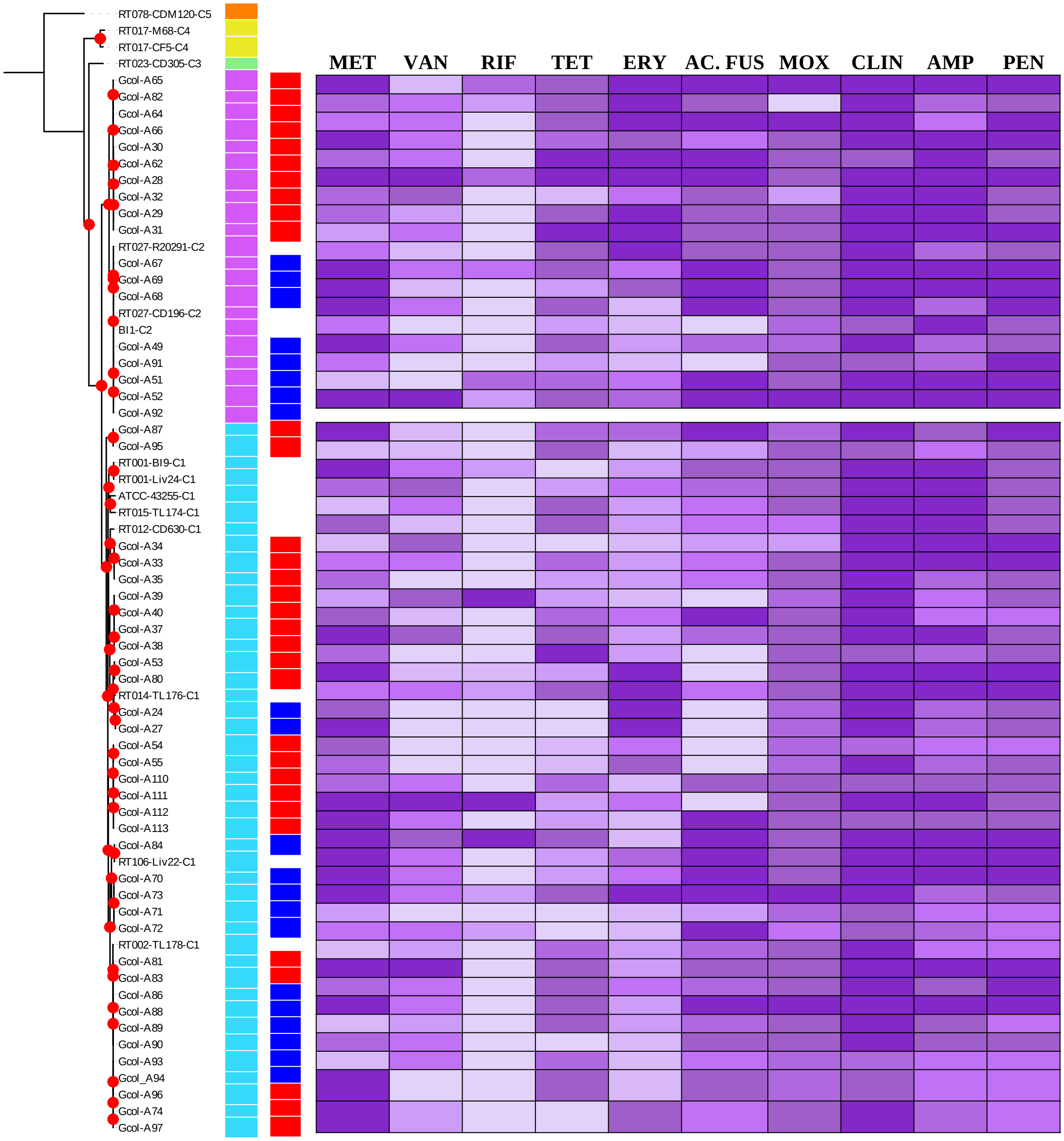

	MET<br>RIF<br>MOX	VAN<br>TET	ERY	FUS.AC<br>AMP<br>PEN	CLI
	≥ 13	≥ 150	≥ 38	≥ 25	≥ 5
	6.26-12.5	76.0-150	18.76-38.0	12.6-25	2.51-5
	3.14-6.25	37.51-75.99	9.39-18.75	6.26-12.5	1.26-2.50
	1.57-3.13	18.75-37.50	4.70-9.38	3.14-6.25	0.64-1.25
	0.79-1.56	9.39-18.74	2.35-4.69	1.57-3.13	0.32-0.63
	0.40-0.78	4.70-9.38	1.18-2.34	0.79-1.56	0.16-0.31
	≤ 0.39	≤ 4.69	≤ 1.17	≤ 0.78	< 0.15

Supplementary Table S1. Isolates information. The assemblies are available in NCBI project PRJNA551724 (https://www.ncbi.nlm.nih.gov/bioproject/PRJNA551724)

Genome ID	Accession number	Sequence Type	Clade	Date of sample collection	Sample ID	Health-care center	Categorization
Gcol-A53	VIMQ00000000	14	1	Sep-2015	Sample-158	FCS	IH
Gcol-A80	VIMB00000000	14	1	Sep-2015	Sample-158	FCS	IH
Gcol-A54	VIMP00000000	26	1	Sep-2015	Sample-160	FCS	IH
Gcol-A55	VIMO00000000	26	1	Sep-2015	Sample-160	FCS	IH
Gcol-A87	VILV00000000	43	1	Oct-2015	Sample-166	FCS	IH
Gcol-A62	VIMN00000000	41	2	Nov-2015	Sample-171	FCS	IH
Gcol-A81	VIMA00000000	8	1	Nov-2015	Sample-172	FCS	IH
Gcol-A83	VILY00000000	8	1	Nov-2015	Sample-172	FCS	IH
Gcol-A82	VILZ00000000	41	2	Nov-2015	Sample-172	FCS	IH
Gcol-A64	VIMM00000000	41	2	Nov-2015	Sample-173	FCS	IH
Gcol-A65	VIML00000000	41	2	Nov-2015	Sample-173	FCS	IH
Gcol-A66	VIMK00000000	41	2	Nov-2015	Sample-173	FCS	IH
Gcol-A67	VIMJ00000000	1	2	Ene-2016	Sample-174	FCS	CO
Gcol-A68	VIMI00000000	1	2	Ene-2016	Sample-174	FCS	CO
Gcol-A69	VIMH00000000	1	2	Ene-2016	Sample-174	FCS	CO
Gcol-A70	VIMG00000000	149	1	Feb-2016	Sample-175	FCS	CO
Gcol-A71	VIMF00000000	149	1	Feb-2016	Sample-175	FCS	CO
Gcol-A72	VIME00000000	149	1	Feb-2016	Sample-175	FCS	CO
Gcol-A73	VIMD00000000	149	1	Feb-2016	Sample-175	FCS	CO
Gcol-A74	VIMC00000000	8	1	Mar-2016	Sample-177	FCS	IH
Gcol-A96	VILM00000000	8	1	Mar-2016	Sample-177	FCS	IH
Gcol-A97	VILL00000000	8	1	Mar-2016	Sample-177	FCS	IH
Gcol-A95	VILN00000000	43	1	Mar-2016	Sample-177	FCS	IH
Gcol-A37	VIMX00000000	29	1	May-2016	Sample-100	HUMM	IH
Gcol-A38	VIMW00000000	29	1	May-2016	Sample-100	HUMM	IH
Gcol-A39	VIMV00000000	29	1	May-2016	Sample-100	HUMM	IH
Gcol-A40	VIMU00000000	29	1	May-2016	Sample-100	HUMM	IH
Gcol-A24	VINH00000000	2	1	Apr-2016	Sample-83	HUMM	CO
Gcol-A27	VING00000000	2	1	Apr-2016	Sample-83	HUMM	CO
Gcol-A28	VINF00000000	41	2	Apr-2016	Sample-84	HUMM	IH
Gcol-A29	VINE00000000	41	2	Apr-2016	Sample-84	HUMM	IH
Gcol-A30	VIND00000000	41	2	Apr-2016	Sample-84	HUMM	IH
Gcol-A31	VINC00000000	41	2	Apr-2016	Sample-84	HUMM	IH
Gcol-A32	VINB00000000	41	2	Apr-2016	Sample-84	HUMM	IH
Gcol-A33	VINA00000000	48	1	Apr-2016	Sample-86	HUMM	IH
Gcol-A34	VIMZ00000000	48	1	Apr-2016	Sample-86	HUMM	IH

Gcol-A35	VIMY00000000	48	1	Apr-2016	Sample-86	HUMM	IH
Gcol-A93	VILP00000000	8	1	Apr-2016	Sample-178	FCS	CO
Gcol-A84	VILX00000000	42	1	Apr-2016	Sample-178	FCS	CO
Gcol-A86	VILW00000000	8	1	Jul-2016	Sample-179	FCS	CO
Gcol-A88	VILU00000000	8	1	Jul-2016	Sample-179	FCS	CO
Gcol-A89	VILT00000000	8	1	Jul-2016	Sample-179	FCS	CO
Gcol-A90	VILS00000000	8	1	Jul-2016	Sample-179	FCS	CO
Gcol-A94	VILO00000000	8	1	Jul-2016	Sample-179	FCS	CO
Gcol-A49	VIMT00000000	1	2	Aug-2016	Sample-147	HUMM	CO
Gcol-A51	VIMS00000000	1	2	Aug-2016	Sample-147	HUMM	CO
Gcol-A52	VIMR00000000	1	2	Aug-2016	Sample-147	HUMM	CO
Gcol-A91	VILR00000000	1	2	Aug-2016	Sample-147	HUMM	CO
Gcol-A92	VILQ00000000	1	2	Aug-2016	Sample-147	HUMM	CO
Gcol-A110	VILK00000000	26	1	Dec-2016	Sample-205	HUMM	IH
Gcol-A111	VILJ00000000	26	1	Dec-2016	Sample-205	HUMM	IH
Gcol-A112	VILI00000000	26	1	Dec-2016	Sample-205	HUMM	IH
Gcol-A113	VILH00000000	26	1	Dec-2016	Sample-205	HUMM	IH

CO: community-acquired infections; FCS: Fundación Clínica Shaio; HUMM: Hospital Universitario Mayor – Méderi; IH: intra-hospital acquired infections

Supplementary Table S2. Unique genes found in Gcol_A84.

Gene	Non-unique Gene name	Annotation
group_5245	soj_1	Sporulation initiation inhibitor protein soj
group_5246	parB_1	Chromosome-partitioning protein parB
group_5247		Predicted outer membrane protein
group_5248		hypothetical protein
group_5249		hypothetical protein
group_5250		hypothetical protein
group_5251		DNA methylase
group_5252		hypothetical protein
group_5253		hypothetical protein
group_5254		DNA primase (bacterial type)
group_5255		hypothetical protein
group_5256		hypothetical protein
group_5257		Relaxase/Mobilisation nuclease domain
group_5258		Protein of unknown function (DUF3801)
group_5259		hypothetical protein
group_5260	traG	Conjugal transfer protein traG
group_5261		hypothetical protein
group_5262		PrgI family protein
group_5263		hypothetical protein
group_5264		Sortase (surface protein transpeptidase)
group_5265		CHAP domain
group_5266		hypothetical protein
mprA_3		Mycobacterial persistence regulator A
group_5268	drrA_3	Daunorubicin/doxorubicin resistance ATP-binding protein DrrA
group_5269		ABC-type transport system involved in multi-copper enzyme maturation, permease component
group_5270	yycG_2	Sensor histidine kinase YycG
group_5271		RNA polymerase sigma factor RpoE
group_5272	ndoA_1	mRNA interferase EndoA
group_5273		hypothetical protein
group_5274	pinR	Putative DNA-invertase from lambdoid prophage Rac
group_5275		Protein of unknown function (DUF3789)
group_5276		Conjugative transposon protein TcpC
group_5278	regX3_5	Sensory transduction protein regX3
group_5279	walK_4	Sensor protein kinase walK
group_5280		Fluoroquinolones export ATP-binding protein Rv2688c/MT2762
group_5281		Uncharacterized protein conserved in bacteria

group_5282		Uncharacterized protein conserved in bacteria
group_5283		DNA-binding transcriptional activator PspC
group_5284	regX3_6	Sensory transduction protein regX3
group_5285	phoR_8	Alkaline phosphatase synthesis sensor protein phoR
group_5286		FtsX-like permease family
group_5287		Uncharacterized protein conserved in bacteria
lolD_9		Lipoprotein-releasing system ATP-binding protein LolD
group_5289		anaerobic benzoate catabolism transcriptional regulator
group_5290		hypothetical protein
group_5291		hypothetical protein
group_5292		RNA polymerase sigma factor
group_5293		Helix-turn-helix domain
group_5294		hypothetical protein

Supplementary Table S3. Reference genomes information.

Number	Genome name	Clade	Features
1	ATCC_43255	1	More abundant. Includes TcdA + / TcdB + members, but negative for binary toxin. Some non-toxigenic members
2	RT001_BI9		
3	RT001_Liv24		
4	RT002_TL178		
5	RT012_CD630		
6	RT014_TL176		
7	RT015_TL174		
8	RT106_Liv22		
9	BI1	2	Hypervirulent strains: TcdA+/TcdB +/CDT+
1o	RT027_CD196		
11	RT027_R20291		
12	RT023_CD305	3	Europe: TcdA+/TcdB +/CDT+
13	RT017_CF5	4	TcdB + exclusively (outbreak)
14	RT017_M68		
15	RT078_CDM120	5	More heterogeneous: Humans, animals, food

Supplementary Table S4. Description of the antimicrobial agents evaluated during the susceptibility tests of the isolates in characterization process.

Antimicrobial agent	MIC range	Concentration considered as resistance	Pharmacological group	Mechanisms of action and resistance	References
Metronidazole	≤0.5–2	0.125–0.6	Nitromidazol	Antibiotic and antiparasitic that causes the breakdown of DNA, the destabilization of the double helix of bacterial DNA and inhibit synthesis of nucleic acids, inhibiting the adequate synthesis of nucleic acids; it is used for the treatment of infections caused by protozoa and anaerobic bacteria; As a mechanism of resistance, possible alterations of metabolic pathways and the formation of biofilms	17-21
Vancomycin	≤0.25–1	0.5 –5	Glycopeptide	This antibiotic, Inhibits the peptidoglycan synthesis, through the inhibition of the enzymes that make up the biosynthetic route of peptidoglycan of the bacterial cell wall; As possible mechanisms of resistance, mutations have been described in the enzymes of the peptidoglycan synthesis pathway, alterations in the antibiotic target proteins (MurG) and the formation of biofilms	17,20-22
Tetracicline	≤0.25–>16		Tetraciclines	The inhibition of bacterial protein synthesis (limiting its growth) has been described as a mechanism of action. Currently, this antimicrobial agent is not widely used in the treatment of C. difficile infections due to the resistance events generated. As a possible mechanism of resistance, the transference of the tetM, tetW and tet(44) genes, which are responsible for the synthesis of ribosome protection proteins	20,21,23-25
Erythromycin	≤0.25–>4		Macrolides	These antimicrobials inhibit protein synthesis in the bacteria, occasionally causing cell death; The transfer of non-conjugative mobile elements (*Tn5398, Tn6194, Tn6215*), which carry copies of the *ermB* gene that encodes a 23S RNA methylase and induces resistance (this has been described as a mechanism of resistance)	20,21,26
Rifampin	≤0.5–>4	0.0039–0.0157	Ansamicinas	As an action mechanism, this antimicrobial agent has been described as a transcription inhibitor, by binding to a central segment (residues 500-580) of the β subunit of the DNA-dependent RNA polymerase. It has activity mainly on Gram-positive microorganisms; however, it can also act on Gram-negative, besides having antiviral activity. As a mechanism of resistance, has been described that mutations in *rpoB* (a gene that codes for the β subunit of bacterial RNA polymerase)	17,20,21,27,28
Ampicillin	≤0.5–4	1–4	β-lactam, penicillin	This antimicrobial limit the biosynthesis of the bacterial cell wall, through the inhibition of peptidoglycan synthesis; It has bactericidal activity in both Gram positive and Gram negative. As a mechanism of resistance, genes have been identified within the genome of C. difficile that encode β-lactamase-type proteins and penicillin-binding proteins (PBP), which	17,20,21,29,30

				would be generating the mechanisms of resistance	
Penicilin	0.5–8	1–4	β-lactam, penicillin	This antibiotic inhibits the biosynthesis of the cell wall of the bacteria. Its activity is mainly on Gram positive bacteria. The mechanisms of resistance are similar to those described for ampicillin	17,20,21,29,30
Fusidic Acid	0.032->256		Cephalosporin	Fusidic acid acts by inhibiting bacterial protein synthesis by blocking the elongation factor G (FE-G), preventing it from binding to the ribosomes and GTP (guanosine triphosphate), thus interrupting the energy supply for the synthesis process. As a mechanism of resistance, mutations have been described in *fusA*, a gene responsible for the coding of FE-G, a ribosomal translocation enzyme	21,31-33
Clindamycin	≤0.25–>8	2-8	Lincosamide	As a mechanism of action, the inhibition of bacterial protein synthesis has been described. As for erythromycin, the transfer of the *ermB* gene has been described as a mechanism of resistance	17,20,21
Moxifloxacin	1–>4	1–4	Fluoroquinolone	This antimicrobial is a powerful new generation synthetic agent widely used in human infections; Its mechanism of action consists in the inhibition of DNA gyrase in Gram negative and topoisomerase IV in Gram positive. Mutations in the quinolone resistance determinant region of the *gyrA* and *gyrB* genes have been decried as resistance mechanisms	17,20,21,24,30,34

Supplementary Data set S1. Assemblies' details of the CD genomes of Colombian isolates.

(Excel File)

References

1 Wattam, A. R. *et al.* PATRIC, the bacterial bioinformatics database and analysis resource. *Nucleic acids research* **42**, D581-591, doi:10.1093/nar/gkt1099 (2014).

2 Wattam, A. R. *et al. Improvements to PATRIC, the all-bacterial Bioinformatics Database and Analysis Resource Center. PATRIC 3.5.4. Search criteria: Genomes/Clostridium* <https://www.patricbrc.org/view/GenomeList/?keyword(clostridium)#view_tab=genomes&filter=eq(genome_status,%22Complete%22)> (2017).

3 Silvester, N. *et al.* The European Nucleotide Archive in 2017. *Nucleic acids research* **46**, D36-D40, doi:10.1093/nar/gkx1125 (2018).

4 Gualtero, S. M. *et al.* [Characteristics of Clostridium difficile infection in a high complexity hospital and report of the circulation of the NAP1/027 hypervirulent strain in Colombia]. *Biomedica : revista del Instituto Nacional de Salud* **37**, 466-472, doi:10.7705/biomedica.v37i4.3244 (2017).

5 Quast, C. *et al.* The SILVA ribosomal RNA gene database project: improved data processing and web-based tools. *Nucleic acids research* **41**, D590-596, doi:10.1093/nar/gks1219 (2013).

6 Page, A. J. *et al.* Robust high-throughput prokaryote de novo assembly and improvement pipeline for Illumina data. *Microbial genomics* **2**, e000083, doi:10.1099/mgen.0.000083 (2016).

7 Zerbino, D. R. & Birney, E. Velvet: algorithms for de novo short read assembly using de Bruijn graphs. *Genome research* **18**, 821-829, doi:10.1101/gr.074492.107 (2008).

8 Boetzer, M., Henkel, C. V., Jansen, H. J., Butler, D. & Pirovano, W. Scaffolding pre-assembled contigs using SSPACE. *Bioinformatics* **27**, 578-579, doi:10.1093/bioinformatics/btq683 (2011).

9 Boetzer, M. & Pirovano, W. Toward almost closed genomes with GapFiller. *Genome biology* **13**, R56, doi:10.1186/gb-2012-13-6-r56 (2012).

10 Figueras, M. J., Beaz-Hidalgo, R., Hossain, M. J. & Liles, M. R. Taxonomic affiliation of new genomes should be verified using average nucleotide identity and multilocus phylogenetic analysis. *Genome announcements* **2**, doi:10.1128/genomeA.00927-14 (2014).

11 Ihaka, R. & Gentleman, R. R: A Language for Data Analysis and Graphics. *Journal of Computational and Graphical Statistics* **5**, 299-314, doi:10.2307/1390807 (1996).

12 Iraola, G. Taxxo v1.0: an R package for integrating and automatising tools for prokaryotes taxonogenomics. *Full user manual*, 1-16 (2017).

13 Page, A. J. *et al.* Comparison of classical multi-locus sequence typing software for next-generation sequencing data. *Microbial genomics* **3**, e000124, doi:10.1099/mgen.0.000124 (2017).

14 Jolley, K. A. & Maiden, M. C. BIGSdb: Scalable analysis of bacterial genome variation at the population level. *BMC bioinformatics* **11**, 595, doi:10.1186/1471-2105-11-595 (2010).

15 Griffiths, D. *et al.* Multilocus sequence typing of Clostridium difficile. *Journal of clinical microbiology* **48**, 770-778, doi:10.1128/JCM.01796-09 (2010).

16 Hunt, M. *et al.* ARIBA: rapid antimicrobial resistance genotyping directly from sequencing reads. *Microbial genomics* **3**, e000131, doi:10.1099/mgen.0.000131 (2017).
17 Patel, J. B. *et al. Performance Standards for Antimicrobial Susceptibility Testing; Twenty-Fourth Informational Supplement*. Vol. 33 (2014).
18 Tenover, F. C., Tickler, I. A. & Persing, D. H. Antimicrobial-resistant strains of Clostridium difficile from North America. *Antimicrob Agents Chemother* **56**, 2929-2932, doi:10.1128/AAC.00220-12 (2012).
19 Moura, I. *et al.* Multidisciplinary analysis of a nontoxigenic Clostridium difficile strain with stable resistance to metronidazole. *Antimicrob Agents Chemother* **58**, 4957-4960, doi:10.1128/AAC.02350-14 (2014).
20 Pirs, T. *et al.* Antimicrobial susceptibility of animal and human isolates of Clostridium difficile by broth microdilution. *J Med Microbiol* **62**, 1478-1485, doi:10.1099/jmm.0.058875-0 (2013).
21 Peng, Z. *et al.* Update on Antimicrobial Resistance in Clostridium difficile: Resistance Mechanisms and Antimicrobial Susceptibility Testing. *Journal of clinical microbiology* **55**, 1998-2008, doi:10.1128/JCM.02250-16 (2017).
22 Leeds, J. A., Sachdeva, M., Mullin, S., Barnes, S. W. & Ruzin, A. In vitro selection, via serial passage, of Clostridium difficile mutants with reduced susceptibility to fidaxomicin or vancomycin. *J Antimicrob Chemother* **69**, 41-44, doi:10.1093/jac/dkt302 (2014).
23 Donhofer, A. *et al.* Structural basis for TetM-mediated tetracycline resistance. *Proc Natl Acad Sci U S A* **109**, 16900-16905, doi:10.1073/pnas.1208037109 (2012).
24 Spigaglia, P. Recent advances in the understanding of antibiotic resistance in Clostridium difficile infection. *Ther Adv Infect Dis* **3**, 23-42, doi:10.1177/2049936115622891 (2016).
25 Tsutsumi, L. S., Owusu, Y. B., Hurdle, J. G. & Sun, D. Progress in the discovery of treatments for C. difficile infection: A clinical and medicinal chemistry review. *Curr Top Med Chem* **14**, 152-175 (2014).
26 Farrow, K. A., Lyras, D. & Rood, J. I. Genomic analysis of the erythromycin resistance element Tn5398 from Clostridium difficile. *Microbiology* **147**, 2717-2728, doi:10.1099/00221287-147-10-2717 (2001).
27 Yurieva, O., Nikiforov, V., Jr., Nikiforov, V., O'Donnell, M. & Mustaev, A. Insights into RNA polymerase catalysis and adaptive evolution gained from mutational analysis of a locus conferring rifampicin resistance. *Nucleic Acids Res* **45**, 11327-11340, doi:10.1093/nar/gkx813 (2017).
28 O'Connor, J. R. *et al.* Rifampin and rifaximin resistance in clinical isolates of Clostridium difficile. *Antimicrob Agents Chemother* **52**, 2813-2817, doi:10.1128/AAC.00342-08 (2008).
29 Brook, I. Treatment of anaerobic infection. *Expert Rev Anti Infect Ther* **5**, 991-1006, doi:10.1586/14787210.5.6.991 (2007).
30 Hooper, D. C. Mechanisms of action of antimicrobials: focus on fluoroquinolones. *Clin Infect Dis* **32 Suppl 1**, S9-S15, doi:10.1086/319370 (2001).
31 Borg, A. *et al.* Fusidic acid targets elongation factor G in several stages of translocation on the bacterial ribosome. *J Biol Chem* **290**, 3440-3454, doi:10.1074/jbc.M114.611608 (2015).

32 Noren, T., Akerlund, T., Wullt, M., Burman, L. G. & Unemo, M. Mutations in fusA associated with posttherapy fusidic acid resistance in Clostridium difficile. *Antimicrob Agents Chemother* **51**, 1840-1843, doi:10.1128/AAC.01283-06 (2007).

33 Noren, T., Alriksson, I., Akerlund, T., Burman, L. G. & Unemo, M. In vitro susceptibility to 17 antimicrobials of clinical Clostridium difficile isolates collected in 1993-2007 in Sweden. *Clin Microbiol Infect* **16**, 1104-1110, doi:10.1111/j.1469-0691.2009.03048.x (2010).

34 Ackermann, G. *et al.* Resistance to moxifloxacin in toxigenic Clostridium difficile isolates is associated with mutations in gyrA. *Antimicrob Agents Chemother* **45**, 2348-2353, doi:10.1128/AAC.45.8.2348-2353.2001 (2001).
